# Atrial Fibrillation and Long QT Syndrome Presenting in a 12-Year-Old Girl

**DOI:** 10.1155/2012/124838

**Published:** 2012-11-05

**Authors:** Jonathan W. Knoche, Kate M. Orland, Craig T. January, Kathleen R. Maginot

**Affiliations:** ^1^Department of Pediatrics, Mayo School of Graduate Medical Education, 200 First Street SW, Rochester, MN 55905, USA; ^2^Division of Cardiovascular Medicine, Department of Medicine, University of Wisconsin School of Medicine and Public Health, H4/5 Clinical Science Center, 600 Highland Avenue, Madison, WI 53792, USA; ^3^Division of Cardiology, Department of Pediatrics, University of Wisconsin School of Medicine and Public Health, H6/5 Clinical Science Center, 600 Highland Avenue, Madison, WI 53792, USA

## Abstract

Atrial fibrillation (AF) is rare in the pediatric population; however, there is increasing recognition that AF can be inherited. Long QT syndrome (LQTS), likewise, can be both acquired and inherited with mutations leading to abnormalities in cardiac ion channel function. Mutations in KCNQ1 are the most common cause of LQTS. Although rare, mutations in KCNQ1 also can cause familial AF. This report describes a child with a KCNQ1 missense mutation who uniquely expresses concomitant AF and LQTS. Due to the potential for increased morbidity and mortality, young patients who present with AF and a family history suggestive of inherited arrhythmias should trigger further investigation for LQTS and subsequent familial genetic counseling.

## 1. Introduction

Atrial fibrillation (AF) is the most common clinical arrhythmia, affecting 9% of people by age 80 [1]. However, the prevalence of AF in children is rare, occurring in 0.1% of the population [2]. AF is commonly an acquired disorder associated with hypertension, hyperthyroidism, and cardiac structural disease. However, AF can be a heritable condition. Like AF, long QT syndrome (LQTS) may be either acquired or inherited. LQTS results in abnormalities in ventricular repolarization that can cause torsade de pointes which may lead to syncope, seizures, or sudden death. Currently there are 13 known genes associated with LQTS; the majority of LQTS mutations alter potassium or sodium ion channel function [3]. Phenotypic variation within families is common. Although patients with very prolonged repolarization on baseline ECG appear to be in a higher risk for cardiac events [4], sudden death events can occur in patients with normal repolarization on baseline ECG [5]. Current guidelines for genotype positive LQTS patients with a prolonged QTc on baseline ECG recommend beta-blocker therapy, avoidance of drugs that prolong repolarization, and restrictions from most competitive athletics [6].

## 2. Case Report

**Figure 1:**
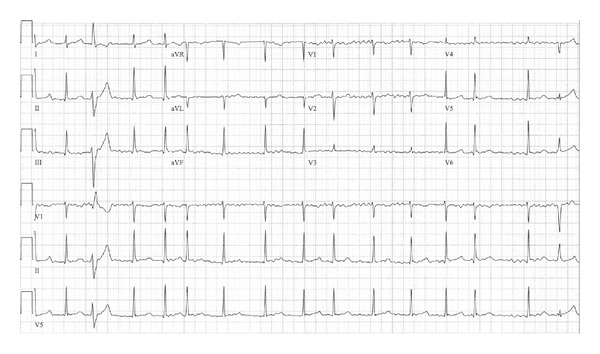
ECG at presentation revealing atrial fibrillation and intermittent premature ventricular contractions. Ventricular rate of 65–150 bpm.

A 12-year-old girl presented with a four-month history of progressive dizziness, fatigue, and intermittent headache. She reported decreased exercise tolerance while participating in competitive volleyball and cheerleading. The patient denied symptoms of palpitations, syncope, or chest pain. Cardiac examination was notable only for an irregularly irregular rhythm of 65–150 bpm. Her weight was 63.5 kg, and blood pressure was 104/57 mmHg. She had clear lung fields, no hepatomegaly, and no edema. Her ECG showed AF (Figure 1). Echocardiogram showed a structurally normal heart with mild left ventricular dilation and mildly decreased biventricular systolic function with no atrial enlargement. Thyroid hormone levels were within normal limits. She was started on Coumadin. Antiarrhythmic medications were not initiated. A Holter monitor showed persistent AF with heart rates ranging from 49–206 bpm, average 97 bpm, and intermittent single premature ventricular contractions.

**Figure 2:**
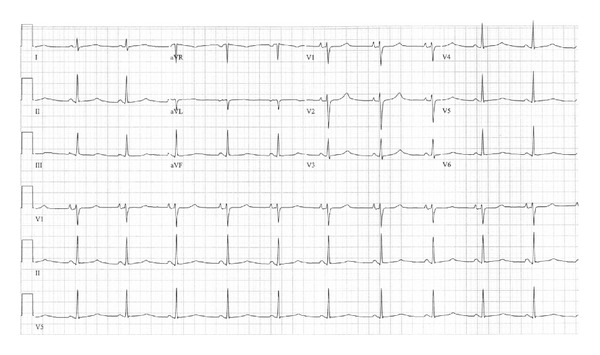
ECG obtained after AF cardioversion, showing sinus rhythm with QTc of 500 ms.

After one month of anticoagulation, the patient underwent a transesophageal echocardiogram and elective synchronized DC cardioversion. Her postconversion ECG showed sinus rhythm at 70 bpm with a rate corrected QT interval (QTc) of 500 ms (Figure 2). She later underwent an exercise stress test that revealed a peak QTc of 520 ms and no exercise-induced arrhythmias.

**Figure 3:**
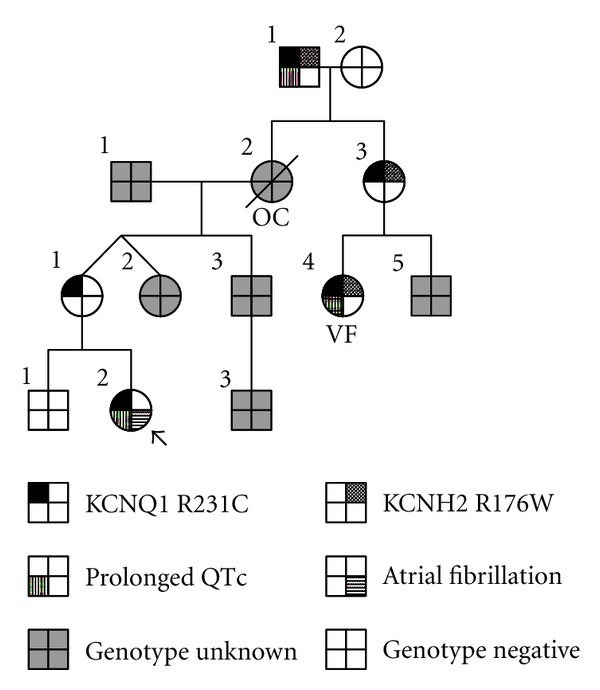
Family pedigree. Index patient (arrow). Ventricular fibrillation (VF). Obligate carrier (OC).

The family history (Figure 3) revealed that the mother's cousin had previously been diagnosed with LQTS, after presenting with ventricular fibrillation. Genetic testing of this cousin revealed two LQTS-associated mutations. These were single nucleotide changes resulting in the missense mutations Arg-231-Cys (R231C) in KCNQ1 and Arg-176-Trp (R176W) in KCNH2 (hERG). KCNQ1 encodes the pore- forming *α*-subunits of the I_Ks_ potassium channel responsible for the slowly activating delayed rectifier potassium current in cardiomyocytes. Disease-causing mutations in KCNQ1 are the most common inherited form of LQTS (long QT syndrome type 1 or LQT1), and cardiac events tend to occur during exertion [7]. Mutations in KCNH2 cause long QT syndrome type 2 (LQT2) and are the second most common cause of LQTS. The KCNH2 gene encodes the *α*-subunits for the rapidly activating delayed rectifier potassium channel, I_Kr_. LQT2 patients often have cardiac events associated with emotional triggers and auditory stimuli [7]. Our patient's mother was asymptomatic, and her ECG showed normal sinus rhythm and a normal QTc. She was positive for the same KCNQ1 mutation and negative for the KCNH2 mutation, giving her a genetic diagnosis of LQT1. The maternal grandmother died six months postpartum at age 27, reportedly due to a pulmonary embolus, with no genetic information available. She is an obligate carrier for the KCNQ1 mutation. Genetic testing on our patient was positive for the KCNQ1 mutation, and her brother was found to be negative. 

The patient was started on low-dose beta-blocker therapy (nadolol 10 mg daily). However, the patient began experiencing frequent nonexertional presyncope, fatigue, and intermittent headaches. Holter and event monitor recordings showed sinus rhythm and sinus bradycardia with no tachyarrhythmias. Repeat echocardiography showed normalization of ventricular systolic function and chamber size. Her symptoms of lightheadedness were suggestive of neurally-mediated presyncope, and she was asked to increase her fluid and sodium intake. She was eventually started on fludrocortisone, which failed to improve her symptoms.

Due to continued episodes of presyncope, the patient was changed to a beta-1 selective agent, atenolol, which failed to improve her symptoms. She expressed a strong interest in restarting competitive athletics. Following lengthy conversations of therapeutic options, an implantable cardioverter defibrillator (ICD) was implanted. At six months of followup, she was participating in competitive athletics on beta-blocker medication with markedly improved symptoms, possibly secondary to treatment of bradycardia with atrial pacing. She had no documented atrial or ventricular tachyarrhythmias, and had received no ICD therapies.

## 3. Discussion

The key finding in this report is the coexistence of AF in a young patient with LQT1. Like the congenital forms of LQTS, ion channelopathies have been associated with familial AF and may contribute to its early onset [8, 9]. Chen and coworkers discovered a KCNQ1 mutation, Ser-140-Gly, in a Chinese family that was associated with AF [10], although other studies have not linked AF to KCNQ1 mutations [11]. Recently the LQT1 mutation R231C was reported in six families presenting with AF, fetal bradycardia, or LQTS, where it caused a reduction in I_Ks_ [12]. Interestingly, their patients with AF had normal QTc measurements in contrast to our patient who manifested both AF and LQT1. While a loss of I_Ks_ may account for the pathophysiologic mechanism for prolonged ventricular repolarization in LQT1, it remains mechanistically unclear how the mutation is associated with AF. 

Interestingly, our family's pedigree indicates that the KCNQ1 and KCNH2 mutations segregate on separate alleles. Close scrutiny of the pedigree raises questions regarding the grandmother's death. While she is an obligate carrier of the KCNQ1 mutation, it is unclear whether the grandmother was also a carrier of the KCNH2 mutation. The maternal grandmother died in the postpartum period when patients with KCNH2 mutations are at higher risk for sudden death events [13].

To date, relatively little attention has focused on the relationship between AF and LQTS. A recent report estimates that up to 2% of patients with genetically proven LQTS can have early-onset (age < 50 years) AF [9]. This incidence is higher than the prevalence of AF in the young (1 : 1,000) or congenital LQTS in the overall population (1 : 2,500). Even though the combination of AF and LQTS is uncommon, the possibility of their coexistence should lead to appropriate evaluation, particularly since many pharmacological therapies for AF are contraindicated in patients with LQTS. One study reported a young patient with AF and LQTS treated with amiodarone who suffered severe ventricular tachyarrhythmias requiring multiple external defibrillator therapies, emergent sternotomy, and extracorporeal membrane oxygenation [9]. Due to potential morbidity and mortality, young patients with AF warrant careful scrutiny for ECG repolarization abnormalities and for concerning family histories.

## 4. Conclusion

In the family we report, the index patient has a mixed phenotype of AF and LQT1. Although AF is uncommon in young healthy patients with structurally normal hearts, patients with early onset AF and a family history suggestive of inherited arrhythmias should trigger further evaluation and consideration for genetic testing. These findings may direct appropriate medical management to reduce morbidity and mortality.
